# Measuring Connections Between Children and Pets: Development of the Child–Dog Engagement Scale and Child–Cat Engagement Scale

**DOI:** 10.3390/ani15131845

**Published:** 2025-06-22

**Authors:** Deanna L. Tepper, Tiffani J. Howell, Pauleen C. Bennett

**Affiliations:** Anthrozoology Research Group, School of Psychology and Public Health, La Trobe University, Bendigo, VIC 3552, Australia; t.howell@latrobe.edu.au (T.J.H.); pauleen.bennett@latrobe.edu.au (P.C.B.)

**Keywords:** pet–owner relationships, child–pet relationships, child development, pet care, pet engagement

## Abstract

As more families expand to include pets, it is important to explore how children interact and engage with them. While the past literature has focused on children’s involvement in animal-assisted therapy or their relationships with assistance animals, their everyday relationships with pets remain understudied. Following expert discussion and focus groups, a sample of 319 parents/guardians completed a questionnaire on their child’s relationship with their pet. Analysis of the data resulted in the development of a 38-item Child–Dog Engagement Scale (C-DES) and a 29-item Child–Cat Engagement Scale (C-CES). Each of these measures three factors: Emotional Attachment, Engagement, and Challenges. The development of parallel child–dog and child–cat scales offers a way to measure children’s relationships across these species, while accounting for the different needs of these animals and the unique ways that children may interact and care for them. The scales therefore provide a foundation for future research exploring how children’s relationships with both cats and dogs may contribute to their development.

## Simple Summary

Many parents acquire a pet specifically for their children, and yet, comparative to other types of human–animal relationships, specific interactions between children and pets remain underexplored. The aim of this study was to create contemporary measures of child–pet relationships that use caregiver reports to capture the nuances of how children interact with cats and dogs. We developed the Child–Cat Engagement Scale and Child–Dog Engagement Scale, which measure three components of child–pet relationships: Emotional Attachment, Engagement, and Challenges.

## 1. Introduction

Globally, pet ownership is becoming increasingly common. In Australia, in 2022, approximately 69% of all households reported living with a companion animal (hereafter referred to as a pet), an increase from the 61% reported in 2016 and 2019, prior to the COVID-19 pandemic [1]. Similar statistics have been reported in the United States of America (66%) [2] and the United Kingdom (62%) [3], while the pet industry has been developing rapidly in China [4], South Korea, India, and the Philippines [5,6,7]. The reasons for owning a pet vary, with studies suggesting pet ownership reflects a general love of animals, desire for companionship, and an opportunity to provide nurturance [8,9].

For parents, there may be additional reasons to acquire a pet, with pets being broadly seen as “good for children” [10]. Yet, despite the inclusion and importance of pets in human families, research in this field has primarily focused on children’s interactions with assistance and therapy animals [11,12]. Of the existing literature, cross-sectional research has supported positive relationships between children’s interactions with pets and their socioemotional development, particularly the development of prosocial behaviors and empathy [13,14]. These findings have also been supported by both Australian and English longitudinal studies, suggesting that pet ownership in childhood is associated with stronger prosocial behaviors [15,16], earlier language development [16], and fewer peer problems [15].

While research focusing on child–pet relationships has grown, much of the existing literature has focused on dogs, or “pets” as a broad, undifferentiated category. However, this overlooks the potential for differences in how children are attached to, and engage with, different pet species. Cats, in particular, are commonly kept as pets and differ from dogs in important ways. While cats may be perceived as being less affectionate with children or less willing to be held by a child in comparison to dogs [17], more research is needed to explore child–cat relationships, as, to date, the existing literature is mixed; some research in the literature has found that children’s attachment to pet cats is similar to their attachment to dogs [18,19], while other research has suggested that this is dependent on the age of the child [20], age of the cat [17,21], or how many cats are in the home [17].

Ultimately, further research is needed to explore child–cat relationships and the potential benefits of such relationships. Additionally, for both cats and dogs, more research is needed to examine how children choose to engage and interact with them. As previously discussed, children’s interactions with pets may improve their socioemotional development [13,14], and it has been theorized that children’s engagement in pet care contributes towards such development [22]. That is, pet ownership likely elicits conversations between parents and children regarding pet care, perspective-taking, and the pet’s emotional and mental states. In turn, this may improve theory of mind and empathy, as children learn to understand and anticipate their pet’s non-verbal behavior and needs [22].

Children’s involvement in pet care has been explored in the past, but findings are inconsistent. For example, Davis et al. [23] found that, for school-aged children, pet care is a shared parent–child chore, while for children of a similar age, Fifield and Forsyth [24] suggested that mothers are the main pet caregivers. Limited to dog ownership, Davis [25] found that children are more likely to be involved in recreational activities, such as playing with a dog, giving the dog treats, and teaching the dog new tricks, while mothers participated in more nurturance-based care. More recently, a study by Kerry-Moran and Barker [26] suggested that children are most involved in exercising the dog and cleaning up after the dog outside, while other dog care activities (e.g., bathing and grooming, training) were mostly completed by the mother; no data were collected on who was responsible for providing daily food and water. Overall, it has been acknowledged that the literature on pet caretaking is outdated [27] and, as the last thirty years have seen significant global social and political changes (e.g., an increase in women working full time, a shift away from nuclear families, the recognition of diverse family structures, and the impacts of COVID-19) [28,29,30], further research is needed to explore how children interact with their pets.

It is also important to consider that a child’s relationship with a pet cat or dog may not always be positive, and there may be times when the behavior of the pet, or the responsibility of providing care, may be overwhelming [31,32]. This has been considered in the development of adult–pet measures [33,34], wherein a successful relationship with one’s pet is maintained only when the benefits of that relationship outweigh the costs, or when there is a balance between the benefits and costs. That child–pet relationships may, at times, be viewed negatively has been underexplored in children, other than in a recent paper describing development of a new measure of child–dog relationships, which includes a component called “friction with pet” [35]. As a negative relationship between children and pets may be a reason that parents relinquish pets [36], it is important to explore this further, across species other than dogs.

The primary aim of the current study was to create two new measures exploring child–pet relationships, specific to pet dogs and cats. While other child–pet measures exist, many were developed more than three decades ago, and may no longer fully capture the nuances of contemporary child–pet interactions [31,37], while adapting adult-based measures for children [38,39] may overlook developmental differences and the unique ways that children engage with pets. While at least one contemporary measure exists for assessing children’s relationships with dogs (see Kerns et al. [35]), we wanted to develop comparable species-specific scales, sensitive to the likelihood that children’s engagement in pet care would be at least partially dependent on the species’ care requirements. In recognition that little is known about children’s caregiving behaviors and how it develops or changes over time [40], the age range of the children was kept broad. Finally, as research has largely focused on Australia, the United States, and the United Kingdom, we aimed to recruit participants from other countries, as well as these, to broaden our understanding of child–pet relationships.

## 2. Materials and Methods

This project was approved by the La Trobe University Human Research Ethics Committee (Approval Number: HEC23427). The project consisted of two stages: the initial development of the questionnaires and then analysis of data collected from participants. To develop a set of initial items for the scales, we adapted steps recommended by Carpenter [41] and Zhou [42], as summarized in Table 1.

### 2.1. Phase 1: Development of the Questionnaire

#### 2.1.1. Literature Review

We first conducted a review of existing human–animal relationship scales, including the Pet Attachment Scale—Parent Report [37], Lexington Attachment to Pets Scale [43], Cat/Dog Relationship Scale [33,34], and Pet-Dog Relationship Inventory [35]. We also reviewed the past literature on children’s engagement in pet care [23,24,25,26,44]. This led to the development of 66 unique items, each measured on a 5-point Likert scale. Some of the items (*n* = 12) were believed to encompass both pet species, while others were developed to be specific to dog (*n* = 29) or cat ownership (*n* = 25).

#### 2.1.2. First Expert Discussion

Eight academic colleagues from the La Trobe University Anthrozoology Research Group (anthrozoologyresearchgroup.com; accessed on 1 February 2024), who have backgrounds in psychology, zoology, veterinary science, and dog training, reviewed each item. Based on discussion with this group, one item was reworded (“This pet gets too rough during play” was changed to “My child gets too rough with the pet during play”).

#### 2.1.3. Focus Group Discussion

To further refine the items, we recruited parents to participate in focus groups to discuss their children’s interactions with pets. These focus groups were advertised via Facebook between 30 June and 10 July 2024. As an AUD 25 financial incentive was offered, participants were recruited only after completing an expression of interest form hosted using the QuestionPro web-based survey platform (Seattle, WA, USA; questionpro.com; accessed on 10 July 2024), which allowed us to screen for bots and fake participants. Twelve individuals were contacted with further information about the focus groups. Based on past research on focus group saturation, it was estimated that two focus groups would be sufficient to determine whether the item pool accurately reflected the child–pet relationship, across multiple animal species [45,46]. The final sample size was eight participants, equally distributed across two groups. All participants were women, with children aged between 5- and 17-years. Collectively, the participants owned both dogs and cats, but also guinea pigs, rats, fish, spiny stick insects, a turtle, a bird, and a pony. No further demographic data were collected.

The focus groups met virtually via Zoom for between 50- and 70-min, with each participant using both video and audio. Participants were asked, “As parents, how do you know that your child feels close to your pet?”, “Are there any aspects to having a pet that your child finds challenging?”, and “What pet care responsibilities does your child engage in?” Based on these interviews, one additional item was added to the item pool on general pet ownership (*n* = 13 items total), capturing that children enjoy talking about their pets. The wording of an existing question was also adjusted, to reflect that children may both appropriately and inappropriately display physical affection to their pets.

#### 2.1.4. Second Expert Discussion

For a final review, a second discussion was held with the Anthrozoology Research Group. All items were reviewed, and no further adjustments were made.

### 2.2. Phase 2: Preliminary Validation of the Questionnaire

The outcome of Phase 1 was a questionnaire comprised of 67 unique items probing parent perceptions of their child’s relationship with a pet. The items included some that were shared across pet species (*n* = 13). The remainder were species-specific, with branching logic used to direct participants to relevant questions based on their response to an initial question about species. For example, “My child helps with obedience training [pet name], including informal training at home (e.g., teaching [pet name] to sit)”, was presented to participants answering questions about a dog, but not to participants answering questions about a cat. Similarly, “My child cleans the litter box”, was specific to participants reporting on cat ownership.

The survey also included a small number of questions to check for participant attention and congruence across items. For example, “Please select ‘Rarely’ to show you are paying attention to this question”. The full questionnaire can be found in the Supplementary Materials (File S1: Full questionnaire). It was digitized using QuestionPro and hosted via Prolific Academic (prolific.com; accessed on 21 October 2024), an online research platform where users can participate in research for a small momentary compensation (*M* = GBP £6.81 per h). Screeners were put in place to ensure that participants had both children aged between 4 and 14, and at least one pet.

#### 2.2.1. Participants

Per Tabachnick and Fidell, a minimum of 100 participants was recommended for the chosen data analysis [47]. The final sample size was 319 parent participants, who were predominantly female (*n* = 189; 59.2%), followed by male (*n* = 128; 40.1%) and non-binary/genderfluid (*n* = 2; 0.6%). The mean age was 37.19 years (*SD* = 8.68 years). The participants’ country of birth represented 46 countries across Europe, North America, Africa, Oceania, Asia, and South America. The majority were from the United Kingdom (*n* = 100; 31.30%), the United States (*n* = 70; 21.90%), and South Africa (*n* = 42; 13.2%), with additional representation from Canada (*n* = 12; 3.8%) and Australia (*n* = 10; 3.1%). Participants mostly identified as having an English, Irish, Scottish and/or Welsh (*n* = 106; 33.2%), African (*n* = 57; 17.9%), or European (*n* = 49; 15.4%) background. Many participants had at least an undergraduate degree (*n* = 120; 37.6%) and were employed in full-time work (*n* = 230; 72.1%), and most participants reported their annual income as average (*n* = 158; 49.5%) or above average (*n* = 107; 33.5%) as compared to their country.

#### 2.2.2. Participant Demographics

The first section of the survey asked about participant demographics (e.g., year of birth, gender, educational level, number of children). The second section asked about their child’s demographics (e.g., year of birth, gender, birth order, whether the child lives in the household full time, presence of a disability). The participants were then asked to complete the remaining survey on the pet that their child helped take care of the most and were requested to type the name of this pet in a space provided. This name was used to automatically personalize the remainder of the survey, with [Pet name] being replaced in each question. For example, for a participant who reported that their pet’s name was Bella, the item, “My child considers [Pet name] to be one of their best friends”, was replaced with, “My child considers Bella to be one of their best friends”. Finally, participants were asked to provide the top three reasons for why they acquired that pet.

#### 2.2.3. Child–Pet Engagement Scale

The questionnaire included 13 questions about general pet ownership, measured on 5-point Likert scales (1 = Definitely not true; 5 = Definitely true), which were presented to all participants (e.g., “My child loves [pet name]” and “It would be upsetting for my child if [pet name] died”). Following this, the participants answered species-specific questions on either dogs (*n* = 29 items) or cats (*n* = 25 items). These items were also measured on 5-point Likert scales (1 = Almost never; 5 = Always or Almost always). Additional questions on other pet species (e.g., fish, small mammals, birds) were included in the questionnaire, but are not discussed in this paper.

#### 2.2.4. Pet-Dog Relationship Inventory (PDRI)

Participants who chose a dog as the focal pet that their child engaged with most also completed the PDRI [35]. This measure was chosen as it provides a contemporary analysis of the child–dog relationship, with strong internal reliability across the subscales, Cronbach’s *α* = 0.72 to 0.85. The overall quality of the child’s relationship with their pet dogs was measured using the parent-report version of the PDRI, a 30-item measure of the child–dog relationship. This measure assesses affection, companionship, nurturance, emotional support, friction, and substitution for people. Items were measured on 5-point Likert scales (1 = Almost always true; 5 = Almost never true). In the current study, there was also strong internal reliability, with Cronbach’s *α* = 0.78 to 0.91.

#### 2.2.5. Procedure

Data collection commenced at 11 a.m. 11 October 2024 Australian Eastern Daylight Time (AEDT) and was completed at 8 p.m. on 21 October 2024 AEDT. To capture participants across different time zones, the survey was activated and deactivated regularly throughout the data collection period. A total of 422 eligible individuals commenced the survey. Incomplete entries (*n* = 46) were removed and, after further data cleaning, seven additional participants were excluded for failing the attention checks and/or providing incongruent information. Finally, 50 participants were removed as they provided information about species other than dogs and cats.

#### 2.2.6. Data Analysis

Data were imported from QuestionPro to the Statistical Package for Social Sciences (SPSS; version 29.0.2.0) program for analysis. Preliminary data were manually screened as per Pallant (pp. 55–86) [48]. Prior to our main analyses, descriptive analyses were conducted on the demographic information provided by the participants and the PDRI subscales were calculated as per Kerns et al. [35].

For the new questionnaires, seven items were reverse-scored so that higher scores consistently reflected greater affection towards the pet. Data were then split by species in SPSS, so that the general pet ownership questions were analyzed alongside the species-specific questions separately for dog and cat owners. The suitability of items for data reduction was reviewed using exploratory factor analysis (EFA). For items on dog and cat ownership, all assumptions for factor analysis were met, including the presence of multiple coefficients above 0.3, Kaiser–Meyer–Olkin values above 0.6, and with Bartlett’s Test of Sphericity reaching statistical significance. However, given the presence of some skewness in the data, principal axis factoring was chosen as a robust extraction method. Scree plots were visually inspected for breaks and checked against simulations from the Monte Carlo PCA for Parallel Analysis program. Three components were identified for each species and used to create subscales by summing the items and dividing by the number of items in the subscale. A “total” subscale was also created for both the dog and cat scales, by summing the mean score from each subscale.

Using multiple regression analyses, we explored whether children’s age, gender, birth order, and presence of a disability predicted subscales created for dogs and cats. Correlational analyses were also conducted between the dog-specific data and PDRI subscales.

## 3. Results

The children were aged between 4 and 14 years, with a relatively equal gender distribution. The parents reported that 18 (5.6%) of the children had a disability, with attention-deficit/hyperactivity disorder (*n* = 4; 1.3%) the most common diagnosis. There was a similar distribution between participants who reported that their child primarily engaged with a dog (*n* = 167; 52.4%) versus cat (*n* = 152; 47.6%), with further demographics presented in Table 2.

The data, separated by pet species, were subjected to EFAs using principal axis factoring and oblimin rotation, as the underlying factors were expected to be correlated. An initial analysis of the dog responses found eight components with eigenvalues above 1, with a cumulative percentage of variance explained of 65.69. A visual inspection of the scree plot suggested that four factors would be appropriate for dog–child relationships, while the Monte Carlo analysis suggested three factors would be appropriate. Similarly, an initial analysis of the cat data found seven components with eigenvalues above 1, while the scree plot suggested three, and the Monte Carlo analysis suggested four factors would be appropriate to reduce data. For both the dog and cat responses, both a three- and four-factor analysis was conducted, suppressing small coefficients with an absolute value below 0.4 [47]. Results of the EFAs for both species suggested that a three-factor solution was the best fit, as seen in Table 3 and Table 4. For both species, the factors were labelled “Emotional Attachment”, “Engagement” and “Challenges”. The two separate scales, each comprised of three subscales, were labelled Child–Dog Engagement Scale (C-DES) and Child–Cat Engagement Scale (C-CES), respectively.

For dogs, 38 of the 42 original items were retained, with no further adjustments being made following a reliability analysis, as shown in Table 3. For cats, 30 of the 38 original items were initially retained. However, when exploring the reliability for the cat data, the item “My child takes [Pet name] for visits outside the house (e.g., leash walks [Pet name], visits other family members, school)” was subsequently removed. This decision was based on both the theoretical unsuitability of its inclusion in the “Challenges” factor and its effect on the overall consistency of the subscale; the Cronbach’s alpha improved from 0.32 to 0.74 when this item was removed. As such, the final cat-only scale contained 29 items, as shown in Table 4. The final items, including instructions, administration and scoring, can be found in the Supplementary Files (File S2: C-DES and C-CES scoring and administration). Frequencies and percentages for responses to the final items are also provided in the Supplementary Files (File S3: Frequency tables).

Across the three subscales for the Child–Dog Engagement Scale, the internal consistency was high for Emotional Attachment (Cronbach’s α = 0.92; *M* = 4.27; *SD* = 0.59), Engagement (Cronbach’s α = 0.94; *M* = 3.31; *SD* = 0.80), and Challenges (Cronbach’s α = 0.77; *M* = 4.12; *SD* = 0.76). A total score was calculated for the Child–Dog Engagement Scale, by summing the mean scores for the three subscales. The total scale ranged from 7.97 to 15 (*M* = 11.70; *SD* = 1.43). Internal consistency was high (Cronbach’s α = 0.94).

Across the three subscales for the Child–Cat Engagement Scale, the internal consistency was also high for Emotional Attachment (Cronbach’s α = 0.91; *M* = 4.09; *SD* = 0.58), Engagement (Cronbach’s α = 0.92; *M* = 3.10; *SD* = 0.88), and Challenges (Cronbach’s α = 0.74; *M* = 4.26; *SD* = 0.69). A total score was also calculated for the Child–Cat Engagement Scale, ranging from 7.82 to 14.44 (*M* = 11.45; *SD* = 1.43), and internal consistency was high (Cronbach’s α = 0.92).

To explore the impact of children’s age, gender, birth order, and the presence of a disability on their relationship with their pet, multiple regression analyses were conducted. When exploring children’s Emotional Attachment with their dog, the overall model was not significant, *F* (7, 159) = 1.43, *p* = 0.20, however, child age (11–14 years) did emerge as a predictor, *B* = 0.36, *SE* = 0.13, *β* = 0.25, *t* = 2.85, *p* = 0.005.

The model for Engagement with a dog, *F* (7, 159) = 0.79, *p* = 0.60, was not significant, and nor was the model for Challenges significant, *F* (7, 159) = 0.50, *p* = 0.84. For cats, the overall model was not significant for Emotional Attachment, *F* (8, 143) = 1.14, *p* = 0.34, Engagement, *F* (8, 143) = 1.24, *p* = 0.28, or Challenges, *F* (8, 143) = 1.92, *p* = 0.06, however, child age (7–10 years) did emerge as a predictor, *B* = 0.29, *SE* = 0.14, *β* = 0.19, *t* = 2.05, *p* = 0.043.

Finally, for the dog participants, we explored correlations between the Emotional Attachment, Engagement, and Challenges subscales and the six PDRI subscales. As shown in Table 5, except for friction, the Emotional Attachment scale was consistently positively correlated with the PDRI subscales. The Engagement scale also showed significant positive correlations, with the strong association observed with the PDRI Nurturance subscale. The Challenges subscale was largely uncorrelated with the PDRI subscales, except for a small positive correlation with the PDRI Affection subscale, and a moderate negative correlation with the PDRI Friction subscale. The Total score on the Child–Dog Engagement Scale was positively correlated with all PDRI subscales, apart from PDRI Friction, which was negatively correlated.

## 4. Discussion

The main aim of this study was to create new measures of child–pet relationships. Through exploratory factor analyses, two separate scales were created, exploring child–dog relationships (Child–Dog Engagement Scale; C-DES) and child–cat relationships (Child–Cat Engagement Scale; C-CES), both of which are collectively referred to under the broader title of the Child–Pet Engagement Scale. For both scales, the child–pet relationship encompassed three categories: Emotional Attachment, Engagement, and Challenges. Overall, the C-DES and C-CES provide internally consistent indices of children’s relationships with pet cats and dogs, as reported by their parents.

While the validity of these new measures is still to be established, and the factor structure has yet to be confirmed using confirmatory factor analysis in an independent sample, the three-factor structure is consistent with previous measures of adult–pet relationships. For example, in the original Monash Dog Owner Relationship Scale (MDORS) [33], and its adaptation, the Cat/Dog Relationship Scale (CDORS) [34], both cat and dog ownership were similarly collapsed into three factors: Perceived Emotional Closeness, Perceived Costs, and Cat/Dog–Owner Interactions. In comparison, specific to children, the Pet Dog Relationship Inventory (PDRI) [35] has a six factor structure, although we note that our Dog-Emotional Attachment subscale strongly correlated with PDRI subscales measuring affection, companionship and emotional support. This suggests that this subscale measures similar aspects of the child–pet relationship and supports the convergent validity of our measure.

Interestingly, there were high mean scores for Emotional Attachment and Challenges, for both cats and dogs. As all items in the Challenges factor were reverse-scored, it indicates that parents believe that their children have high levels of emotional closeness with their pet, with relatively few challenges in that relationship. This is consistent with adult–pet relationship measures such as the MDORS, CDORS and DORS28 [33,34,49], which typically report high levels of emotional closeness and relatively low challenges or perceived costs. While the human–pet relationship may have negative aspects associated with burden of care, financial costs, and grief related with bereavement [50], it broadly appears that the perceived positives of these relationships outweigh or mitigate the challenges, for both adults [49] and children. However, it should be considered that the final C-DES and C-CES Challenges subscales only contained four items on Challenges, and that some of the challenges or costs associated with the child–pet relationship may have been missed. For example, while we explored whether the child is too rough with their pet during play, we did not explore intentional acts of cruelty [51,52]. We also did not explore whether the child has ever been injured by the pet, but as a negative child–pet relationship may explain why pets are relinquished [36], this is important to explore further [53].

While the mean scores for Engagement were lower across both species compared to the mean for the other subscales, it still appeared that children moderately engaged in pet care activities. It is possible that these relatively lower scores may indicate that children are not interested in engaging in pet care activities, or that parents are sometimes reluctant to let their child take responsibility for pet care [54]. It is recommended that future research continues to explore how children interact with their pets, and how such activities and responsibilities are shared within families [54]. Future iterations of the C-DES and C-CES would also benefit from developing a child-perspective measure, capturing Emotional Attachment, Engagement and Challenges, to enrich our understanding of the child–pet relationship further.

We explored how child age, gender, birth order, and disability may predict the relationship that children have with their pet. Interestingly, the models were not significant. This was surprising, as age was kept broad in this study and, for non-pet related chores and household activities, older children typically take on more responsibilities [44]. Additionally, it appears that there may be a gender difference in children’s chore engagement, with parents reporting that girls participate in household chores more frequently than boys [44]. However, past literature on how age may impact children’s relationship or engagement with a pet is not consistent. For example, Martin et al. [55] found that playing with a pet was negatively related to age while, more broadly, Muldoon et al. [56] suggested that children’s attachment to their pets decreased with age. In comparison, Charmaraman et al. [27] found no significant decrease in children’s engagement with a pet across age.

A strength of this study is that we recruited through Prolific, allowing us to collect data from a broad and diverse participant pool. Previous literature is often limited through the use of convenience sampling, which may overrepresent individuals with strong emotional connections to their pets, and overrepresent women and participants from Australia, the United Kingdom, and the United States [57,58]. Our study reduced this self-selection bias without eliminating it entirely, as participants still opted to take part in a pet-related study. While our sample was highly educated, which may affect generalizability, most participants reported that their income was average for their country. In addition, our study had a relatively even gender distribution and included numerous participants from South Africa. As cultural differences may shape how people perceive, interact and form attachments to animals [59], this study provides new insights into children’s cross-cultural relationships with pets, which may help future research explore the socioemotional benefits of pet ownership.

As more pets are welcomed not only into human households but also into human families, it is important to continue exploring the relationships that children have with these animals. The development of the C-DES and C-CES provides researchers with the opportunity to continue exploring child–pet relationships. There is increasing interest in the benefits of pet ownership for children, with some research suggesting that children’s pet ownership is associated with improved socioemotional development [13,14,15,16], but little is known about how children chose to interact with their pets. As such, the C-DES and C-CES provide the opportunity to explore Emotional Attachment, Engagement, and Challenges alongside children’s development and socio-emotional outcomes. Additionally, while the C-DES and C-CES have yet to be explored longitudinally, the development of these measures will allow researchers to explore how children’s engagement with their pet changes over time, and how such engagement and attachment is associated with developmental trajectories. As highlighted by Reider et al. [22], interacting with and providing care for an animal requires perspective-taking and the use of theory of mind, which may aid in the development of empathy and prosocial behaviors. Furthermore, it has been proposed that engagement in pet care may improve executive functions [60,61], which are cognitive skills associated with planning, problem solving, and decision making [62]. As such, it is recommended that future research examines the relationship between the C-DES and C-CES, and children’s empathy, prosocial behaviors, and cognitive skills. Furthermore, while the C-DES and C-CES have not been developed for clinical purposes, there is also the potential for these measures to be adapted to explore how children use their pets for support or emotional regulation, to guide conversations around motivation and responsibility, and to explore whether children would benefit from animal-assisted therapy programs.

## 5. Conclusions

By developing parallel child–dog and child–cat scales, this study offers a way to compare children’s relationships across these species, and a way for future research to explore the benefits of such relationships. In addition to exploring emotional attachment, these scales offer a way to examine children’s engagement in pet care and the challenges of these relationships, for which the literature is outdated or non-existent. Future research would benefit from further validating the scales in diverse populations, as well as exploring how children interact with pets beyond cats and dogs, which will contribute to the understanding of how pets can benefit children’s socioemotional and cognitive development.

## Figures and Tables

**Table 1:** Steps involved in questionnaire development.

Step	Nature of Activity	Methods	Number of Items at the End of Step	Item Change
I	Development of construct	Literature review	66	-
II	Development of construct	First expert discussion	66	Rewording of 1 item
III	Development of construct	Focus group discussion	67	Addition of 1 item, rewording of 1 item
IV	Establishment of content validity	Second expert discussion	67	-
V	Establishment of construct validity	Factor analysis	38 (C-DES) and 29 (C-CES) ^1^	Creation of Child–Dog Engagement Scale (C-DES) and Child–Cat Engagement Scale (C-CES).

^1^ = final C-DES and C-CES numbers include 11 identical items that encompass general pet ownership.

**Table 2:** Children’s demographics across focal pet type, as reported by their parents.

	Dog Owners		Cat Owners	
Demographics	*n*	*%*	*n*	*%*
Child age (years)				
4–6	69	41.3	70	46.1
7–9	48	28.7	36	23.7
10–12	34	20.4	36	23.7
13–14	16	9.6	10	6.6
Child gender				
Male	86	51.5	80	52.6
Female	80	47.9	69	45.4
Non-binary/genderqueer/gender-fluid	0	0.0	1	0.7
Prefer not to say	1	0.6	2	1.3
Child’s birth order				
Youngest	39	23.4	36	23.7
Middle	13	7.8	10	6.6
Oldest	79	47.3	50	32.9
Only child	36	21.6	54	35.5
Prefer not to say	0	0.0	2	1.3
Child lives in participant’s household fulltime				
Yes	161	96.4	150	98.7
No	6	3.6	2	1.3
Child has a disability				
Yes	12	7.2	6	3.9
No	154	92.2	143	94.1
Prefer not to say	1	0.6	3	2.0

**Table 3:** Factor loadings and descriptive statistics derived from the three-factor exploratory factor analysis for children’s engagement with pet dogs.

Dog-Emotional Attachment		Dog-Engagement		Dog-Challenges	
Items	Factor Loadings	Items	Factor Loadings	Items	Factor Loadings
I have observed many special moments between my child and [Pet name]	0.824	My child cleans up inside after [Pet name]	0.840	My child feels that [Pet name] stops them from doing things they want to do (e.g., visiting friends, travelling) ^a^	0.850
My child likes to be near [Pet name] when relaxing (e.g., reading, watching TV)	0.756	When necessary, my child gives [Pet name] medicine/vitamins	0.807	My child feels overwhelmed by [Pet name]’s care needs ^a^	0.765
When my child is away from [Pet name] for an extended time, they miss them	0.744	My child cleans up outside after [Pet name]	0.783	My child finds it difficult to balance their time between [Pet name] and other activities (e.g., school, after school events, visiting friends) ^a^	0.722
It would be upsetting for my child if [Pet name] died	0.734	My child takes [Pet name] outside for toilet breaks	0.764	My child gets too rough with [Pet name] during play or when showing affection ^a^	0.416
My child loves [Pet name]	0.697	My child washes or bathes [Pet name]	0.752		
My child feels like [Pet name] helps them through tough times	0.693	My child provides water for [Pet name]	0.701		
My child considers [Pet name] to be one of their best friends	0.690	My child helps with obedience training [Pet name], including informal training at home (e.g., teaching [Pet name] to sit)	0.691		
My child feels happy when interacting with [Pet name]	0.647	My child takes [Pet name] for visits outside the house (e.g., to other family members, school)	0.689		
My child likes to talk about [Pet name] with me and/or other people	0.626	My child notices if [Pet name] is hungry or thirsty	0.677		
My child likes to show appropriate physical affection towards [Pet name] (e.g., gentle pats, hugs, or kisses)	0.625	My child brushes or grooms [Pet name]	0.666		
My child wishes they could always be with [Pet name]	0.586	My child makes sure [Pet name] is safe when visitors are in the house or are around the pet	0.666		
My child thinks [Pet name] is just a pet ^a^	0.497	My child takes [Pet name] for walk/exercise	0.664		
My child believes [Pet name] understands their [my child’s] feelings or emotions	0.496	My child provides food for [Pet name]	0.633		
		My child gives [Pet name] treats	0.612		
		My child helps with teaching [Pet name] new tricks	0.604		
		When necessary, my child goes to the veterinarian with [Pet name]	0.558		
		My child notices if [Pet name] is sick	0.526		
		My child takes photos or videos of [Pet name]	0.507		
		My child checks that [Pet name] is safe in bed or settled at night	0.504		
		[Pet name] sleeps with my child at night	0.461		
		My child is respectful of [Pet name]’s space (e.g., approaching calmly, giving the animal plenty of room)	0.450		

^a^ = item was reverse-scored. The possible range for all subscales is 1–5.

**Table 4:** Factor loadings and descriptive statistics derived from the three-factor exploratory factor analysis for children’s engagement with pet cats.

Cat-Emotional Attachment		Cat-Engagement		Cat-Challenges	
Items	Factor Loadings	Items	Factor Loadings	Items	Factor Loadings
I have observed many special moments between my child and [Pet name]	0.835	My child provides water for [Pet name]	0.973	My child feels overwhelmed by [Pet name]’s care needs ^a^	0.727
My child likes to talk about [Pet name] with me and/or other people	0.753	My child notices if [Pet name] is sick	0.969	My child finds it difficult to balance their time between [Pet name] and other activities (e.g., school, after school events, visiting friends) ^a^	0.607
My child considers [Pet name] to be one of their best friends	0.750	My child provides food for [Pet name]	0.926	My child feels that [Pet name] stops them from doing things they want to do (e.g., visiting friends, travelling) ^a^	0.606
When my child is away from [Pet name] for an extended time, they miss them	0.745	When necessary, my child gives [Pet name] medicine/vitamins	0.822	[Pet name]’s behaviour (or noise) bothers or irritates my child ^a^	0.508
My child likes to be near [Pet name] when relaxing (e.g., reading, watching TV)	0.738	When necessary, my child goes to the veterinarian with [Pet name]	0.815		
My child loves [Pet name]	0.722	My child cleans up after [Pet name]	0.631		
My child wishes they could always be with [Pet name]	0.687	My child gives [Pet name] treats	0.547		
My child feels like [Pet name] helps them through tough times	0.632	My child notices if [Pet name] is hungry or thirsty	0.484		
My child believes [Pet name] understands their [my child’s] feelings or emotions	0.597	My child cleans the litter box ^b^	0.410		
My child likes to show appropriate physical affection towards [Pet name] (e.g., gentle pats, hugs, or kisses)	0.529				
It would be upsetting for my child if [Pet name] died	0.523				
My child checks that [Pet name] is safe in bed or settled at night	0.504				
My child entertains or plays with [Pet name]	0.497				
[Pet name] sleeps with my child at night	0.492				
My child feels happy when interacting with [Pet name]	0.479				
My child makes sure [Pet name] is safe when visitors are in the house or are around the pet	0.468				

^a^ = item was reverse-scored, ^b^ = item cross-loaded with the “Challenges” component, but loaded more strongly on the “Engagement” component. The possible range for all subscales is 1–5.

**Table 5:** Correlational analyses for three Child–Dog Engagement Scale subscales and total scale, and six PDRI subscales.

	Pet-Dog Relationship Inventory					
	Affection	Companionship	Nurturance	Emotional Support	Friction	Substitute
**Child–Dog Engagement Scale**						
Emotional Attachment	0.80 **	0.77 **	0.68 **	0.78 **	−0.26 **	0.63 **
Engagement	0.50 **	0.67 **	0.79 **	0.66 **	0.00	0.70 **
Challenges	0.16 *	0.02	0.01	−0.04	−0.49 **	−0.18 *
Total	0.70 **	0.70 **	0.72 **	0.67 **	−0.37 **	0.55 **

* *p* < 0.05, ** *p* < 0.01, █ = large correlation, █ = medium correlation, █ = small correlation.

## Data Availability

Data collected for this study are available on request from the corresponding author, provided that approval to release the data is obtained from the La Trobe University Human Ethics Committee.

## Supplementary Materials

The following supporting information can be downloaded at: https://www.mdpi.com/article/10.3390/ani15131845/s1, File S1: Full questionnaire; File S2: C-DES and C-CES scoring and administration. File S3: Frequency tables.
