# Supplementary material for: Measuring Connections Between Children and Pets: Development of the Child–Dog Engagement Scale and Child–Cat Engagement Scale
Source: Animals (Basel). 2025 Jun 22;15(13):1845. doi: 10.3390/ani15131845 (PMC12248443; doi:10.3390/ani15131845)
Supplement: Supplementary file 1 [file animals-15-01845-s001.zip › File S1 - Survey 22.06.2025.pdf]

File S1: Survey

**Exploring children's relationship and attachment to pets and how this predicts their executive functions; parents' and guardians' view**

This research is being carried out in partial fulfilment of a Doctor of Philosophy (PhD) degree under the supervision of Professor Pauleen Bennett. The following researchers will be conducting the study:

- **Main Supervisor:** Prof Pauleen Bennett
- **Co-Supervisor:** Dr Tiffani Howell
- **Student Researcher:** Ms Deanna Tepper

This research is supported by in kind support by La Trobe University.

**1. What is the study about?**

You are invited to participate in a study exploring children's attachment and interactions with pets. This study is part of a larger PhD project. Although it is apparent that pets are important to children, there are some gaps in our understanding of how children interact with pets, particularly across different animal studies. More research is also needed to explore how children's relationship with pets might benefit the development of memory and self-control skills.

**2. Do I have to participate?**

Being part of this study is voluntary. If you want to be part of the study we ask that you read the information below carefully. You can read the information below and decide at the end if you do not want to participate. If you decide not to participate this won't affect your relationship with La Trobe University.

**3. Who is being asked to participate?**

You have been asked to participate because:

- You are at least 18 years of age;
- You have a child aged between 5- and 13-years of age;
- You reside with a pet or pets in your household;
- You understand written English

**4. What will I be asked to do?**

If you want to take part in this study, we will ask you to rate statements about your child's relationship with a pet. If you have more than one pet, we ask that your report on the pet that your child helps take care of the most. We will also ask you to rate statements about your child's memory and self-control skills. This survey will take approximately 15 minutes of your time.

**5. What are the benefits?**

The expected benefit to society is to understand how interacting with benefits may be used to effectively help children develop cognitive skills. There will be no individual benefits of participating in this study, although some people find it rewarding to contribute to new knowledge.

**6. What are the risks?**

With any study there are (1) risks we know about, (2) risks we don't know about and (3) risks we don't expect. We do not foresee any risks associated with this study. However, if you experience something that you aren't sure about, please contact us immediately so we can discuss the best way to manage your concerns.

- Ms Deanna Tepper
- Student Researcher
- d.tepper@latrobe.edu.au

**7. What will happen to information about me?**

By clicking on the 'I agree, start questionnaire' button, this tells us you want to take part in the study.

We will **collect**, **store**, and **publish** information about you in ways that **WILL NOT** reveal who you are. The findings of this study will be used in a PhD thesis and may be published in journal papers or books, summarised for inclusion in newsletters on online, presented at conferences, as well as used for future research projects. In the above publications, you WILL NOT be individually identifiable.

We will **keep** your information for 5 years after the project is completed. During this period, this information may be used for future, related projects. After this time, we will destroy all of your data. The storage, transfer and destruction of your data will be undertaken in accordance with the Research Data Management Policy https://policies.latrobe.edu.au/document/view.php?id=106/.

**8. Will I hear about the results of the study?**

As per the Terms and Conditions of Prolific, we cannot directly collect identifiable information, including your e-mail address. As such, we will send a summary of our findings via the in-built Prolific messaging system after 01 December 2024.

**9. What if I change my mind?**

It is important for you to understand that any data you enter into the survey will be immediately saved and used within the study. If you decide that you no longer want to complete the questionnaire, simply close the web browser any time, knowing that all data entered before that point will be retained. We cannot exclude your data because your responses will be anonymous, so we will not be able to link you with your data. Your decision to withdraw at any point will not affect your relationship with La Trobe University.

**10. Who can I contact for questions or want more information?**

If you would like to speak to us, please use the contact details below:

- Ms Deanna Tepper

- Student Researcher
- d.tepper@latrobe.edu.au

- Prof Pauleen Bennett
- Main Supervisor
- pauleen.bennett@latrobe.edu.au

**11. What if I have a complaint?**

If you have a complaint about any part of this study, please contact:

- Ethics Reference Number: HEC23427
- Senior Research Ethics Officer
- +61 3 9479 1443
- humanethics@latrobe.edu.au

I (the participant) have read and understood the Participant Information Statement, and any questions have been answered to my satisfaction. I agree to participate in the study. I agree information provided by me or with my permission during the project may be included in a thesis, presentation and published in journals on the condition that I cannot be identified.

I am at least 18 years old, the parent or legal guardian of a child aged between 5- to 13-years of age, and can read and write in English.

**I agree, start questionnaire**

**What is your Prolific ID? _____**

**The first few questions are about you, to help us analyse the results from this survey.**

**What is your year of birth?**

2006 - 1900

**How do you describe your gender?**

Male

Female

Non-binary/genderqueer/gender-fluid

Prefer not to say

I use a different term (please specify)

**In which country were you <u>born</u>?**

Afghanistan - Zimbabwe

**In which country do you <u>currently reside</u>?**

Afghanistan - Zimbabwe

**How many adults (18 years or older) currently live in your household?**

1

2

3

4

5 or more

Prefer not to say

**How many children (under 18 years) are you parent or guardian for that live in your household?**

1

2

3

4

5 or more

Prefer not to say

**By participating in this study, you have acknowledged that you are the parent or legal guardian to a child aged between 5- and 13-years of age. <u>If you have more than one child in that age range, please refer to the child who is most responsible for looking after a pet in the home.</u>**

**What is your child's year of birth?**

2020 – 2010

**What is your child's preferred gender identity?**

Male

Female

Non-binary/genderqueer/gender-fluid

Prefer not to say

My child uses a different term (please specify)

**What is this child's birth order in the household?**

Youngest

Middle

Oldest

Only child

Prefer not to say

Other (please specify)

**Does this child live in your household full-time?**

Yes

No

**Does your child have a disability or a condition that impacts on their daily activities, communication and/or mobility, and has lasted or is likely to last 6 months or more?**

Yes

No

Prefer not to say

**How would you describe your child's disability or chronic condition?**
**Select all the apply.**

Attention-deficit/hyperactivity disorder

Autism

Blind or visually impaired

Deaf or hard of hearing

Dyslexia

Health-related disability

Intellectual disability

Mental health condition

Mobility-related disability

Speech-related disability

Other (please specify)

**The next questions are going to ask about your pet. If you have more than one pet, we ask you to please refer to the pet that your child helps take care of the most.**

**What is the name of your pet? _____**

**What type of pet is [pet name]?**
Dog

Cat

Fish

Caged bird (e.g., cockatiel, canary)

Chicken

Small mammal (e.g., rabbit, guinea pig)

Reptile

Horse or livestock (e.g., sheep, alpaca)

Insects or spiders

Other (please specify): _____

**What were the top three (3) reasons why you decided to get [pet name]? Please rank them in order of importance, with one (1) being the most important.**

Drag your choices here to rank them

- For my/my family’s mental health (e.g., sense of purpose, connection)
- Previous experience with family or friends’ pets
- I did not decide to get my pet (e.g., it was given to me, I inherited it)
- Someone to look after/provide a sense of family
- Impact from COVID-19 lockdowns (e.g., spent more time at home, boredom)
- Companionship for another pet
- For work or products (e.g., breeding, farm working dogs, egg-laying)
- To teach my child responsibility/respect/patience
- Spontaneous opportunity (e.g., pet was offered to me, on sale)
- I wanted to help/save the animal
- Fun/entertainment/as a hobby (e.g., sport, showing or competition)
- Security for me/my family
- For my/my family’s physical health (e.g., exercise, walking)
- Companionship for me/my family
- My family member(s) wanted one
- Other

If 'Other', please specify why your family decided to get [pet name]: _____

**Below, you will find a number of statements. Please read each statement carefully and indicate how much you agree with it. You indicate your response by clicking one of the circles after each statement.**

| | Definitely Not True | Not True | Partially True | True | Definitely True |
|---|---|---|---|---|---|
| My child loves [pet name] | | | | | |
| When my child is away from [pet name] for an extended time, they miss them | | | | | |
| My child wishes they could always be with [pet name] | | | | | |
| My child feels like [pet name] helps them through tough times | | | | | |
| My child feels happy when interacting with [pet name] | | | | | |
| My child thinks [pet name] is just a pet | | | | | |
| My child believes [pet name] understands their [my child's] feelings or emotions | | | | | |
| My child considers [pet name] to be one of their best friends | | | | | |
| My child likes to talk about [pet name] with me and/or other people | | | | | |
| It would be upsetting for my child if [pet name] died | | | | | |
| I have observed many special moments between my child and [pet name] | | | | | |
| My child likes to be near [pet name] when relaxing (e.g., reading, watching TV) | | | | | |
| [pet name]'s behaviour (or noise) bothers or irritates my child | | | | | |

--- DOG OWNERS ONLY ---

**Below, you will find a number of statements. Please read each statement carefully and thereafter indicate how much you agree with that statement. You indicate your response by clicking one of the circles after each statement.**

| | Almost Never | Rarely | Sometimes | Very Often | Always or Almost Always |
|---|---|---|---|---|---|
| My child takes photos or videos of [pet name] | | | | | |
| My child likes to show appropriate physical affection towards [pet name] (e.g., gentle pats, hugs, or kisses) | | | | | |
| My child finds it difficult to balance their time between [pet name] and other activities (e.g., school, after school events, visiting friends) | | | | | |
| My child feels overwhelmed by [pet name]'s care needs | | | | | |
| My child feels that [pet name] stops them from doing things they want to do (e.g., visiting friends, travelling) | | | | | |
| My child gets too rough with [pet name] during play or when showing affection | | | | | |
| My child provides food for [pet name] | | | | | |
| My child notices if [pet name] is sick | | | | | |
| When necessary, my child goes to the veterinarian with [pet name] | | | | | |

| | Almost Never | Rarely | Sometimes | Very Often | Always or Almost Always |
|---|---|---|---|---|---|
| When necessary, my child gives [pet name] medicine/vitamins | | | | | |
| Please select 'Rarely' to show you are paying attention to this question | | | | | |
| My child provides water for [pet name] | | | | | |
| My child gives [pet name] treats | | | | | |
| My child notices if [pet name] is hungry or thirsty | | | | | |
| My child takes [pet name] outside for toilet breaks | | | | | |
| My child cleans up inside after [pet name] | | | | | |
| My child cleans up outside after [pet name] | | | | | |
| My child would help look for [pet name] if this pet was lost | | | | | |
| [pet name] sleeps with my child at night | | | | | |
| My child checks that [pet name] is safe in bed or settled at night | | | | | |
| My child makes sure [pet name] is safe when visitors are in the house or are around the pet | | | | | |
| My child helps with obedience training [pet name], including informal | | | | | |

| | Almost Never | Rarely | Sometimes | Very Often | Always or Almost Always |
|---|---|---|---|---|---|
| training at home (e.g., teaching to sit) | | | | | |
| My child helps with teaching [pet name] new tricks | | | | | |
| My child entertains or plays with [pet name] | | | | | |
| My child takes [pet name] for walk/exercise | | | | | |
| My child brushes or grooms [pet name] | | | | | |
| My child washes or bathes [pet name] | | | | | |
| My child takes [pet name] for visits outside the house (e.g., to other family members, school) | | | | | |
| My child is respectful of [pet name]'s space (e.g., approaching calmly, giving the animal plenty of room) | | | | | |
| My child gets annoyed at [pet name] | | | | | |

**Now we are going to ask you some additional questions about your child and your pet. Some of these are very similar to the previous questions, but don't worry if you see some questions that look a lot alike.**

| | Almost Never True | Sometimes True | Often True | True Most of the Time | Almost Always True |
|---|---|---|---|---|---|
| My child loves this pet. | | | | | |
| My child helps take care of the pet. | | | | | |

| | Almost Never True | Sometimes True | Often True | True Most of the Time | Almost Always True |
|---|---|---|---|---|---|
| When my child is upset, being with the pet helps my child feel better. | | | | | |
| This pet can be a nuisance (frustrates child) at times. | | | | | |
| This dog and my child spend a lot of time together. | | | | | |
| This pet means more to my child than any of their friends. | | | | | |
| My child feels happy when around the pet. | | | | | |
| My child pays attention to what the pet needs. | | | | | |
| My pet is aware of how my child feels. | | | | | |
| This pet has habits (does things) that annoy my child. | | | | | |
| This pet and my child play together every day. | | | | | |
| This pet is my child's *very* best friend. | | | | | |
| My child feels fond of (affection for) the pet. | | | | | |
| My child comforts this pet if this pet is upset. | | | | | |
| My child talks to this pet about problems. | | | | | |
| My child get mad at this pet. | | | | | |
| This pet stays near or follows my child around. | | | | | |

| | Almost Never True | Sometimes True | Often True | True Most of the Time | Almost Always True |
|---|---|---|---|---|---|
| This pet is more loyal to my child than anyone else. | | | | | |
| My child feels very close to this pet. | | | | | |
| My child helps do daily chores for this pet like feeding. | | | | | |
| My child finds it comforting to be around this pet. | | | | | |
| This pet gets too rough during play with my child. | | | | | |
| My child and this pet do fun things together. | | | | | |
| My child would rather spend time with this pet than anyone else. | | | | | |
| My child feels devoted to this pet. | | | | | |
| My child is responsible for helping with this pet. | | | | | |
| My child thinks this pet understands him or her. | | | | | |
| This pet's behavior bothers or irritates my child. | | | | | |
| My child likes to have the pet near when studying, reading, or watching TV. | | | | | |
| My child loves this pet more than anyone else. | | | | | |

--- CAT OWNERS ONLY ---

**Below, you will find a number of statements. Please read each statement carefully and thereafter indicate how much you agree with that statement. You indicate your response by clicking one of the circles after each statement**.

| | Almost Never | Rarely | Sometimes | Very Often | Always or Almost Always |
|---|---|---|---|---|---|
| My child takes photos or videos of [pet name] | | | | | |
| My child likes to show appropriate physical affection towards [pet name] (e.g., gentle pats, hugs, or kisses) | | | | | |
| My child finds it difficult to balance their time between [pet name] and other activities (e.g., school, after school events, visiting friends) | | | | | |
| My child feels overwhelmed by [pet name]'s care needs | | | | | |
| My child feels that [pet name] stops them from doing things they want to do (e.g., visiting friends, travelling) | | | | | |
| My child gets too rough with [pet name] during play or when showing affection | | | | | |
| My child provides food for [pet name] | | | | | |
| My child notices if [pet name] is sick | | | | | |
| When necessary, my child goes to the veterinarian with [pet name] | | | | | |

| | Almost Never | Rarely | Sometimes | Very Often | Always or Almost Always |
|---|---|---|---|---|---|
| When necessary, my child gives [pet name] medicine/vitamins | | | | | |
| My child provides water for [pet name] | | | | | |
| My child gives [pet name] treats | | | | | |
| My child notices if [pet name] is hungry or thirsty | | | | | |
| My child cleans the litter box | | | | | |
| Please select 'Rarely' to show you are paying attention to this question | | | | | |
| My child cleans up after [pet name] | | | | | |
| My child would help look for [pet name] if this pet was lost | | | | | |
| [pet name] sleeps with my child at night | | | | | |
| My child checks that [pet name] is safe in bed or settled at night | | | | | |
| My child makes sure [pet name] is safe when visitors are in the house or are around the pet | | | | | |
| My child helps with obedience training [pet name], including informal training at home (e.g., teaching to use scratching post) | | | | | |

| | Almost Never | Rarely | Sometimes | Very Often | Always or Almost Always |
|---|---|---|---|---|---|
| My child entertains or plays with [pet name] | | | | | |
| My child brushes or grooms [pet name] | | | | | |
| My child takes [pet name] for visits outside the house (e.g., leash walks , visits other family members, school) | | | | | |
| My child is respectful of [pet name]'s space (e.g., approaching calmly, giving the animal plenty of room) | | | | | |
| My child gets annoyed at [pet name] | | | | | |

--- FISH OWNERS ONLY ---

Below, you will find a number of statements. Please read each statement carefully and thereafter indicate how much you agree with that statement. You indicate your response by clicking one of the circles after each statement.

| | Almost Never | Rarely | Sometimes | Very Often | Always or Almost Always |
|---|---|---|---|---|---|
| My child takes photos or videos of [pet name] | | | | | |
| My child finds it difficult to balance their time between [pet name] and other activities (e.g., school, after school events, visiting friends) | | | | | |
| My child feels overwhelmed by [pet name]'s care needs | | | | | |
| My child feels that [pet name] stops them from doing things they want to | | | | | |

| | Almost Never | Rarely | Sometimes | Very Often | Always or Almost Always |
|---|---|---|---|---|---|
| do (e.g., visiting friends, travelling) | | | | | |
| My child provides food for [pet name] | | | | | |
| My child notices if [pet name] is sick | | | | | |
| When necessary, my child gives [pet name] medicine/vitamins | | | | | |
| My child gives [pet name] treats | | | | | |
| My child helps clean the fishbowl, tank, aquarium, or pond | | | | | |
| My child helps monitor the water health and quality (e.g., monitor water pH levels) | | | | | |
| Please select 'Rarely' to show you are paying attention to this question | | | | | |
| My child cares for [pet name]'s environment by decorating the fishbowl or aquarium by placing ornaments, rocks or plants (i.e., aqua scaping) | | | | | |
| My child is respectful of [pet name]'s space (e.g., not banging on glass) | | | | | |
| My child gets annoyed at [pet name] | | | | | |

--- BIRD OWNERS ONLY ---

Below, you will find a number of statements. Please read each statement carefully and thereafter indicate how much you agree with that statement. You indicate your response by clicking one of the circles after each statement.

| | Almost Never | Rarely | Sometimes | Very Often | Always or Almost Always |
|---|---|---|---|---|---|
| My child takes photos or videos of [pet name] | | | | | |
| My child likes to show appropriate physical affection towards [pet name] (e.g., gentle pats, kisses) | | | | | |
| My child finds it difficult to balance their time between [pet name] and other activities (e.g., school, after school events, visiting friends) | | | | | |
| My child feels overwhelmed by [pet name]'s care needs | | | | | |
| My child feels that [pet name] stops them from doing things they want to do (e.g., visiting friends, travelling) | | | | | |
| My child provides food for [pet name] | | | | | |
| My child notices if [pet name] is sick | | | | | |
| When necessary, my child goes to the veterinarian with [pet name] | | | | | |
| When necessary, my child gives [pet name] medicine/vitamins | | | | | |
| My child provides water for [pet name] | | | | | |

| | Almost Never | Rarely | Sometimes | Very Often | Always or Almost Always |
|---|---|---|---|---|---|
| Please select 'Rarely' to show you are paying attention to this question | | | | | |
| My child gives [pet name] treats | | | | | |
| My child notices if [pet name] is hungry or thirsty | | | | | |
| My child cleans [pet name]'s cage | | | | | |
| My child would help look for [pet name] if this pet was lost | | | | | |
| My child checks that [pet name] is safe in bed or settled at night | | | | | |
| My child makes sure [pet name] is safe when visitors are in the house or are around the pet | | | | | |
| My child helps with obedience training [pet name], including informal training at home (e.g., familiarising to touch, teaching to 'step up') | | | | | |
| My child helps with helps with teaching [pet name] new tricks (e.g., talking) | | | | | |
| My child entertains or plays with [pet name] | | | | | |
| My child provides enrichment for [pet name] by placing decorations or toys into the cage | | | | | |

| | Almost Never | Rarely | Sometimes | Very Often | Always or Almost Always |
|---|---|---|---|---|---|
| My child is respectful of [pet name]’s space (e.g., not banging on cage) | | | | | |
| My child gets annoyed at [pet name] | | | | | |

--- CHICKEN OWNERS ONLY ---

Below, you will find a number of statements. Please read each statement carefully and thereafter indicate how much you agree with that statement. You indicate your response by clicking one of the circles after each statement.

| | Almost Never | Rarely | Sometimes | Very Often | Always or Almost Always |
|---|---|---|---|---|---|
| My child takes photos or videos of [pet name] | | | | | |
| My child likes to show appropriate physical affection towards [pet name] (e.g., gentle pats, hugs, or kisses) | | | | | |
| My child finds it difficult to balance their time between [pet name] and other activities (e.g., school, after school events, visiting friends) | | | | | |
| My child feels overwhelmed by [pet name]'s care needs | | | | | |
| My child feels that [pet name] stops them from doing things they want to do (e.g., visiting friends, travelling) | | | | | |
| My child provides food for [pet name] | | | | | |

| | Almost Never | Rarely | Sometimes | Very Often | Always or Almost Always |
|---|---|---|---|---|---|
| My child notices if [pet name] is sick | | | | | |
| When necessary, my child goes to the veterinarian with [pet name] | | | | | |
| When necessary, my child gives [pet name] medicine/vitamins | | | | | |
| My child provides water for [pet name] | | | | | |
| Please select 'Rarely' to show you are paying attention to this question | | | | | |
| My child gives [pet name] treats | | | | | |
| My child notices if [pet name] is hungry or thirsty | | | | | |
| My child assists with cleaning the coop | | | | | |
| My child checks that [pet name] is safe in bed or settled at night | | | | | |
| My child makes [pet name] sure is safe when visitors are around the pet | | | | | |
| My child helps collect eggs | | | | | |
| My child is respectful of [pet name]'s space (e.g., approaching calmly, giving the animal plenty of room) | | | | | |
| My child trains, entertains or plays with [pet name] | | | | | |
| My child gets annoyed at [pet name] | | | | | |

--- SMALL MAMMAL OWNERS ONLY ---

Below, you will find a number of statements. Please read each statement carefully and thereafter indicate how much you agree with that statement. You indicate your response by clicking one of the circles after each statement.

| | Almost Never | Rarely | Sometimes | Very Often | Always or Almost Always |
|---|---|---|---|---|---|
| My child takes photos or videos of [pet name] | | | | | |
| My child likes to show appropriate physical affection towards [pet name] (e.g., gentle pats or kisses) | | | | | |
| My child finds it difficult to balance their time between [pet name] and other activities (e.g., school, after school events, visiting friends) | | | | | |
| My child feels overwhelmed by [pet name]'s care needs | | | | | |
| My child feels that [pet name] stops them from doing things they want to do (e.g., visiting friends, travelling) | | | | | |
| My child gets too rough with [pet name] during play or when showing affection | | | | | |
| My child provides food for [pet name] | | | | | |
| My child notices if [pet name] is sick | | | | | |
| When necessary, my child goes to the veterinarian with [pet name] | | | | | |

| | Almost Never | Rarely | Sometimes | Very Often | Always or Almost Always |
|---|---|---|---|---|---|
| When necessary, my child gives [pet name] medicine/vitamins | | | | | |
| Please select 'Rarely' to show you are paying attention to this question | | | | | |
| My child provides water for [pet name] | | | | | |
| My child gives [pet name] treats or pellets | | | | | |
| My child notices if [pet name] is hungry or thirsty | | | | | |
| My child cleans [pet name]'s enclosure | | | | | |
| My child entertains or plays with [pet name] | | | | | |
| My child provides enrichment for [pet name] by placing nesting materials or toys in their enclosure | | | | | |
| My child is respectful of [pet name]'s space (e.g., not banging on enclosure) | | | | | |
| My child gets annoyed at [pet name] | | | | | |

--- REPTILE OWNERS ONLY ---

Below, you will find a number of statements. Please read each statement carefully and thereafter indicate how much you agree with that statement. You indicate your response by clicking one of the circles after each statement.

| | Almost Never | Rarely | Sometimes | Very Often | Always or Almost Always |
|---|---|---|---|---|---|
| My child takes photos or videos of [pet name] | | | | | |
| My child likes to show appropriate physical affection towards [pet name]  (e.g., gentle pats) | | | | | |
| My child finds it difficult to balance their time between [pet name]and other activities (e.g., school, after school events, visiting friends) | | | | | |
| My child feels overwhelmed by [pet name]'s care needs | | | | | |
| My child feels that [pet name] stops them from doing things they want to do (e.g., visiting friends, travelling) | | | | | |
| My child provides food for [pet name] | | | | | |
| My child notices if [pet name] is sick | | | | | |
| When necessary, my child goes to the veterinarian with [pet name] | | | | | |
| When necessary, my child gives [pet name] medicine/vitamins | | | | | |
| My child provides water for [pet name] (e.g., refilling water bowls or misting the enclosure) | | | | | |
| Please select 'Rarely' to show you are paying attention to this question | | | | | |

| | Almost Never | Rarely | Sometimes | Very Often | Always or Almost Always |
|---|---|---|---|---|---|
| My child gives [pet name] treats | | | | | |
| My child notices if [pet name] is hungry or thirsty | | | | | |
| My child cleans [pet name]'s enclosure | | | | | |
| My child provides enrichment for [pet name] by providing decorations or arranging hiding spots in their enclosure | | | | | |
| My child monitors the lighting/humidity/temperature for [pet name]'s enclosure | | | | | |
| My child is respectful of [pet name]'s space (e.g., not banging on enclosure) | | | | | |
| My child gets annoyed at [pet name] | | | | | |

--- HORSE/LIVESTOCK OWNERS ONLY ---

Below, you will find a number of statements. Please read each statement carefully and thereafter indicate how much you agree with that statement. You indicate your response by clicking one of the circles after each statement.

| | Almost Never | Rarely | Sometimes | Very Often | Always or Almost Always |
|---|---|---|---|---|---|
| My child takes photos or videos of [pet name] | | | | | |
| My child likes to show appropriate physical affection towards [pet name] | | | | | |

| | Almost Never | Rarely | Sometimes | Very Often | Always or Almost Always |
|---|---|---|---|---|---|
| (e.g., gentle pats, hugs, or kisses) | | | | | |
| My child finds it difficult to balance their time between [pet name] and other activities (e.g., school, after school events, visiting friends) | | | | | |
| My child feels overwhelmed by [pet name]’s care needs | | | | | |
| My child feels that [pet name] stops them from doing things they want to do (e.g., visiting friends, travelling) | | | | | |
| My child provides food for [pet name] | | | | | |
| My child notices if [pet name] is sick | | | | | |
| When necessary, my child goes to the veterinarian with [pet name] | | | | | |
| When necessary, my child gives [pet name] medicine/vitamins | | | | | |
| My child provides water for [pet name] | | | | | |
| Please select ‘Rarely’ to show you are paying attention to this question | | | | | |
| My child gives [pet name] treats | | | | | |
| My child notices if [pet name] is hungry or thirsty | | | | | |

| | Almost Never | Rarely | Sometimes | Very Often | Always or Almost Always |
| --- | --- | --- | --- | --- | --- |
| My child entertains or plays with [pet name] | | | | | |
| My child brushes or grooms [pet name] | | | | | |
| My child cleans [pet name]'s stall or pen | | | | | |
| My child makes sure is safe when visitors are around the pet | | | | | |
| My child is respectful of [pet name]'s space (e.g., approaching calmly, giving the animal plenty of room) | | | | | |
| My child gets annoyed at [pet name] | | | | | |

--- INSECT OWNERS ONLY ---

Below, you will find a number of statements. Please read each statement carefully and thereafter indicate how much you agree with that statement. You indicate your response by clicking one of the circles after each statement.

| | Almost Never | Rarely | Sometimes | Very Often | Always or Almost Always |
|---|---|---|---|---|---|
| My child takes photos or videos of [pet name] | | | | | |
| My child finds it difficult to balance their time between [pet name]and other activities (e.g., school, after school events, visiting friends) | | | | | |
| My child feels overwhelmed by [pet name]'s care needs | | | | | |
| My child feels that [pet name] stops them from doing things they want to do (e.g., visiting friends, travelling) | | | | | |
| My child provides food for [pet name] | | | | | |
| My child notices if [pet name] is sick | | | | | |
| When necessary, my child gives [pet name] medicine/vitamins | | | | | |
| My child provides water for [pet name] (e.g., refilling water bowls, providing a damp sponge, or misting the enclosure) | | | | | |
| My child gives [pet name] treats | | | | | |
| My child notices if [pet name] is hungry or thirsty | | | | | |
| Please select 'Rarely' to show you are paying attention to this question | | | | | |
| My child cleans [pet name]’s enclosure | | | | | |

| | Almost Never | Rarely | Sometimes | Very Often | Always or Almost Always |
|---|---|---|---|---|---|
| My child provides [pet name] enrichment for by providing decorations or arranging hiding spots in their enclosure | | | | | |
| My child monitors the lighting/humidity/temperature for [pet name]'s enclosure | | | | | |
| My child is respectful of [pet name]'s space (e.g., not banging on enclosure] | | | | | |
| My child gets annoyed at [pet name] | | | | | |

--- OTHER ANIMALS ---

Below, you will find a number of statements. Please read each statement carefully and thereafter indicate how much you agree with that statement. You indicate your response by clicking one of the circles after each statement.

| | Almost Never | Rarely | Sometimes | Very Often | Always or Almost Always |
|---|---|---|---|---|---|
| My child takes photos or videos of [pet name] | | | | | |
| My child likes to show appropriate physical affection towards [pet name] (e.g., gentle pats, hugs, or kisses) | | | | | |
| My child finds it difficult to balance their time between [pet name] and other activities (e.g., school, after school events, visiting friends) | | | | | |
| My child feels overwhelmed by [pet name]'s care needs | | | | | |

| | Almost Never | Rarely | Sometimes | Very Often | Always or Almost Always |
|---|---|---|---|---|---|
| My child feels that [pet name] stops them from doing things they want to do (e.g., visiting friends, travelling) | | | | | |
| My child provides food for [pet name] | | | | | |
| My child notices if [pet name] is sick | | | | | |
| When necessary, my child goes to the veterinarian with [pet name] | | | | | |
| When necessary, my child gives [pet name] medicine/vitamins | | | | | |
| My child provides water for [pet name] | | | | | |
| Please select 'Rarely' to show you are paying attention to this question | | | | | |
| My child gives [pet name] treats | | | | | |
| My child notices if [pet name] is hungry or thirsty | | | | | |
| My child helps [pet name] clean where lives | | | | | |
| My child entertains or plays with [pet name] | | | | | |
| My child brushes or grooms [pet name] | | | | | |
| My child is respectful of [pet name]'s space | | | | | |
| My child gets annoyed at [pet name] | | | | | |

Is there anything else you would like to share about your child's relationship or experiences with [pet name]? ___________________________________________________________________________

Below, you will find a number of statements. Please read each statement carefully and thereafter indicate how well that statement is true for **<u>your child</u>**. You indicate your response by clicking one of the circles after each statement.

| | Definitely Not True | Not True | Partially True | True | Definitely True |
|---|---|---|---|---|---|
| Has difficulty remembering lengthy instructions | | | | | |
| Seldom seems to be able to motivate him-/herself to do something that he/she doesn't want to do | | | | | |
| Has difficulty remembering what he/she is doing, in the middle of an activity | | | | | |
| Has difficulty following through on less appealing tasks unless he/she is promised some type of reward for doing so | | | | | |
| Has a tendency to do things without first thinking about what could happen | | | | | |
| When asked to do several things, he/she only remembers the first or last | | | | | |
| Has difficulty coming up with a different way of solving a problem when he/she gets stuck | | | | | |
| When something needs to be done, he/she is often distracted by something more appealing | | | | | |
| Easily forgets what he/she is asked to fetch | | | | | |

| | Definitely Not True | Not True | Partially True | True | Definitely True |
|---|---|---|---|---|---|
| Gets overly excited when something special is going to happen (e.g., going on a field trip, going to a party) | | | | | |
| Please select 'True' to show you are paying attention to this question | | | | | |
| Has clear difficulties doing things he/she finds boring | | | | | |
| Has difficulty planning for an activity (e.g., remembering to bring everything necessary for a field trip or things needed for school) | | | | | |
| Has difficulty holding back his/her activity despite being told to do so | | | | | |
| Has difficulty carrying out activities that require several steps (e.g., for younger children, getting completely dressed without reminders; for older children, doing all homework independently) | | | | | |
| In order to be able to concentrate, he/she must find the task appealing | | | | | |
| Has difficulty refraining from smiling or laughing in situations where it is inappropriate | | | | | |
| Has difficulty telling a story about something that has happened so that others may easily understand | | | | | |
| Has difficulty stopping an activity immediately upon being told to do so. For | | | | | |

| | Definitely Not True | Not True | Partially True | True | Definitely True |
|---|---|---|---|---|---|
| example, he/she needs to jump a couple of extra times or play on the computer a little bit longer after being asked to stop | | | | | |
| Has difficulty understanding verbal instructions unless he/she is also shown how to do something | | | | | |
| Has difficulty with tasks or activities that involve several steps | | | | | |
| Has difficulty thinking ahead or learning from experience | | | | | |
| Acts in a wilder way compared to other children in a group (e.g., at a birthday party or during a group activity) | | | | | |
| Has difficulty doing things that require mental effort, such as counting backwards | | | | | |
| Has difficulty keeping things in mind while he/she is doing something else | | | | | |

**Information about ethnicity, employment and income is very important to understand. To help us analyse the results of this survey, please answer these final questions about yourself.**

**How do you best describe yourself?**

Aboriginal and/or Torres Strait Islander

African

Australian

Central and/or South American

Central Asian

East and/or South-East Asian

English, Irish, Scottish and/or Welsh

European (including Western, Eastern and South-Eastern European, and Scandinavian)

Maori

Middle Eastern

New Zealander

North American

Black or African American

American Indian or Alaska Native

Native Hawaiian or Pacific Islander

South Asian

Prefer not to say

Other (please specify) _____

**Which of the following best describes your <u>current</u> employment status?**

Retired

Unemployed

Unable to work

Engaged in home duties

Part time/casual paid work (30 hours or less per week)

Full time paid work (more than 30 hours per week)

Prefer not to say

Other (please specify) _____

**What is the highest level of formal education that you have completed?**

No formal schooling

Year 10 or below (up to age 16 years)

Year 11 or 12 (above age 16 years)

Vocational or technical training (e.g., Certificate, diploma, advanced diploma, associate degree, technical/trade qualification, TAFE)

Undergraduate degree (e.g., Bachelor’s degree)

Graduate degree (e.g., Master’s degree, PhD)

Prefer not to say

Other (please specify) _____

**How do you perceive your household’s total income (before taxes) from previous years, compared to the average of your country.**

Very Low

Below Average

Average

Above Average

Very High

Prefer not to say
