# Supplementary material for: Measuring Connections Between Children and Pets: Development of the Child–Dog Engagement Scale and Child–Cat Engagement Scale
Source: Animals (Basel). 2025 Jun 22;15(13):1845. doi: 10.3390/ani15131845 (PMC12248443; doi:10.3390/ani15131845)
Supplement: Supplementary file 1 [file animals-15-01845-s001.zip › File S2 - C-DES and C-CES administration and scoring 22.06.2025.pdf]

File S2: C-DES and C-CES scoring and administration

Child-Dog Engagement Scale (C-DES)

Instructions: Below, you will find a number of statements. Please read each statement carefully and thereafter indicate how much you agree with that statement. You indicate your response by clicking one of the circles after each statement.

---

1. My child feels happy when interacting with [Dog name]

Definitely Not True	Not True	Partially True	True	Definitely True
☐	☐	☐	☐	☐

2. My child gives [Dog name] treats

Almost Never	Rarely	Sometimes	Very Often	Always or Almost Always
☐	☐	☐	☐	☐

3. My child takes photos or videos of [Dog name]

Almost Never	Rarely	Sometimes	Very Often	Always or Almost Always
☐	☐	☐	☐	☐

4. When necessary, my child gives [Dog name] medicine/vitamins

Almost Never	Rarely	Sometimes	Very Often	Always or Almost Always
☐	☐	☐	☐	☐

5. My child takes [Dog name] outside for toilet breaks

Almost Never	Rarely	Sometimes	Very Often	Always or Almost Always
☐	☐	☐	☐	☐

6. When my child is away from [Dog name] for an extended time, they miss them

Definitely Not True	Not True	Partially True	True	Definitely True
☐	☐	☐	☐	☐

7. My child likes to be near [Dog name] when relaxing (e.g., reading, watching TV)

Definitely Not True	Not True	Partially True	True	Definitely True
☐	☐	☐	☐	☐

8. [Dog name] sleeps with my child at night

Almost Never	Rarely	Sometimes	Very Often	Always or Almost Always
☐	☐	☐	☐	☐

9. My child loves [Dog name]

Definitely Not True	Not True	Partially True	True	Definitely True
☐	☐	☐	☐	☐

10. When necessary, my child goes to the veterinarian with [Dog name]

Almost Never	Rarely	Sometimes	Very Often	Always or Almost Always
☐	☐	☐	☐	☐

11. My child makes sure [Dog name] is safe when visitors are in the house or are around the pet

Almost Never	Rarely	Sometimes	Very Often	Always or Almost Always
☐	☐	☐	☐	☐

12. My child finds it difficult to balance their time between [Dog name] and other activities (e.g., school, after school events, visiting friends)

Almost Never	Rarely	Sometimes	Very Often	Always or Almost Always
☐	☐	☐	☐	☐

13. My child is respectful of [Dog name]'s space (e.g., approaching calmly, giving the animal plenty of room)

Almost Never	Rarely	Sometimes	Very Often	Always or Almost Always
☐	☐	☐	☐	☐

14. My child feels like [Dog name] helps them through tough times

Definitely Not True	Not True	Partially True	True	Definitely True
☐	☐	☐	☐	☐

15. My child washes or bathes [Dog name]

Almost Never	Rarely	Sometimes	Very Often	Always or Almost Always
☐	☐	☐	☐	☐

16. My child helps with obedience training [Dog name], including informal training at home (e.g., teaching [Dog name] to sit)

Almost Never	Rarely	Sometimes	Very Often	Always or Almost Always
☐	☐	☐	☐	☐

17. My child likes to show appropriate physical affection towards [Dog name] (e.g., gentle pats, hugs, or kisses)

Definitely Not True	Not True	Partially True	True	Definitely True
☐	☐	☐	☐	☐

18. My child cleans up outside after [Dog name]

Almost Never	Rarely	Sometimes	Very Often	Always or Almost Always
☐	☐	☐	☐	☐

19. My child likes to talk about [Dog name] with me and/or other people

Definitely Not True	Not True	Partially True	True	Definitely True
☐	☐	☐	☐	☐

20. My child believes [Dog name] understands their [my child's] feelings or emotions

Definitely Not True	Not True	Partially True	True	Definitely True
☐	☐	☐	☐	☐

21. My child gets too rough with [Dog name] during play or when showing affection

Almost Never	Rarely	Sometimes	Very Often	Always or Almost Always
☐	☐	☐	☐	☐

22. My child considers [Dog name] to be one of their best friends

Definitely Not True	Not True	Partially True	True	Definitely True
☐	☐	☐	☐	☐

23. My child thinks [Dog name] is just a pet

Definitely Not True	Not True	Partially True	True	Definitely True
☐	☐	☐	☐	☐

24. It would be upsetting for my child if [Dog name] died

Definitely Not True	Not True	Partially True	True	Definitely True
☐	☐	☐	☐	☐

25. My child notices if [Dog name] is sick

Almost Never	Rarely	Sometimes	Very Often	Always or Almost Always
☐	☐	☐	☐	☐

26. My child helps with teaching [Dog name] new tricks

Almost Never	Rarely	Sometimes	Very Often	Always or Almost Always
☐	☐	☐	☐	☐

27. My child checks that [Dog name] is safe in bed or settled at night

Almost Never	Rarely	Sometimes	Very Often	Always or Almost Always
☐	☐	☐	☐	☐

28. My child provides food for [Dog name]

Almost Never	Rarely	Sometimes	Very Often	Always or Almost Always
☐	☐	☐	☐	☐

29. My child notices if [Dog name] is hungry or thirsty

Almost Never	Rarely	Sometimes	Very Often	Always or Almost Always
☐	☐	☐	☐	☐

30. My child provides water for [Dog name]

Almost Never	Rarely	Sometimes	Very Often	Always or Almost Always
☐	☐	☐	☐	☐

31. My child takes [Dog name] for walk/exercise

Almost Never	Rarely	Sometimes	Very Often	Always or Almost Always
☐	☐	☐	☐	☐

32. My child wishes they could always be with [Dog name]

Definitely Not True	Not True	Partially True	True	Definitely True
☐	☐	☐	☐	☐

33. I have observed many special moments between my child and [Dog name]

Definitely Not True	Not True	Partially True	True	Definitely True
☐	☐	☐	☐	☐

34. My child takes [Dog name] for visits outside the house (e.g., to other family members, school)

Almost Never	Rarely	Sometimes	Very Often	Always or Almost Always
☐	☐	☐	☐	☐

35. My child feels overwhelmed by [Dog name]'s care needs

Almost Never	Rarely	Sometimes	Very Often	Always or Almost Always
☐	☐	☐	☐	☐

36. My child brushes or grooms [Dog name]

Almost Never	Rarely	Sometimes	Very Often	Always or Almost Always
☐	☐	☐	☐	☐

37. My child feels that [Dog name] stops them from doing things they want to do (e.g., visiting friends, travelling)

Almost Never	Rarely	Sometimes	Very Often	Always or Almost Always
☐	☐	☐	☐	☐

38. My child cleans up inside after [Dog name]

Almost Never	Rarely	Sometimes	Very Often	Always or Almost Always
☐	☐	☐	☐	☐

Administration and Scoring Instructions for the C-DES

Each item is scored on a five-point scale, from 1 to 5. As specified below, five items should be reverse scored, such that a higher score indicates stronger relationships.

To calculate the Emotional Attachment subscale score, reverse score item 23, and then add the scores for items 1, 6, 7, 9, 14, 17, 19, 20, 22, 23, 24, 32, and 33. Then divide by 13.

To calculate the Engagement subscale score, add the scores for items 2, 3, 4, 5, 8, 10, 11, 13, 15, 16, 18, 25, 26, 27, 28, 29, 30, 31, 34, 36, and 38. Then divide by 21.

To calculate the Challenges subscale score, reverse score items 12, 21, 35, and 37. Then add the scores and divide by 4.

To calculate the Total score, sum the three subscale scores.

Child-Cat Engagement Scale (C-CES)

Instructions: Below, you will find a number of statements. Please read each statement carefully and thereafter indicate how much you agree with that statement. You indicate your response by clicking one of the circles after each statement.

---

1. **My child feels happy when interacting with [Cat name]**

Definitely Not True	Not True	Partially True	True	Definitely True
☐	☐	☐	☐	☐

2. **My child gives [Cat name] treats**

Almost Never	Rarely	Sometimes	Very Often	Always or Almost Always
☐	☐	☐	☐	☐

3. **When necessary, my child gives [Cat name] medicine/vitamins**

Almost Never	Rarely	Sometimes	Very Often	Always or Almost Always
☐	☐	☐	☐	☐

4. **When my child is away from [Cat name] for an extended time, they miss them**

Definitely Not True	Not True	Partially True	True	Definitely True
☐	☐	☐	☐	☐

5. **My child likes to be near [Cat name] when relaxing (e.g., reading, watching TV)**

Definitely Not True	Not True	Partially True	True	Definitely True
☐	☐	☐	☐	☐

6. **My child loves [Cat name]**

Definitely Not True	Not True	Partially True	True	Definitely True
☐	☐	☐	☐	☐

7. **When necessary, my child goes to the veterinarian with [Cat name]**

Almost Never	Rarely	Sometimes	Very Often	Always or Almost Always
☐	☐	☐	☐	☐

8. **My child feels like [Cat name] helps them through tough times**

Definitely Not True	Not True	Partially True	True	Definitely True
☐	☐	☐	☐	☐

9. **My child likes to show appropriate physical affection towards [Cat name] (e.g., gentle pats, hugs, or kisses)**

Definitely Not True	Not True	Partially True	True	Definitely True
☐	☐	☐	☐	☐

10. **My child cleans up after [Cat name]**

Almost Never	Rarely	Sometimes	Very Often	Always or Almost Always
☐	☐	☐	☐	☐

11. **My child notices if [Cat name] is sick**

Almost Never	Rarely	Sometimes	Very Often	Always or Almost Always
☐	☐	☐	☐	☐

12. **My child likes to talk about [Cat name] with me and/or other people**

Definitely Not True	Not True	Partially True	True	Definitely True
☐	☐	☐	☐	☐

13. **My child believes [Cat name] understands their [my child's] feelings or emotions**

Definitely Not True	Not True	Partially True	True	Definitely True
☐	☐	☐	☐	☐

14. **My child considers [Cat name] to be one of their best friends**

Definitely Not True	Not True	Partially True	True	Definitely True
☐	☐	☐	☐	☐

15. **My child provides food for [Cat name]**

Almost Never	Rarely	Sometimes	Very Often	Always or Almost Always
☐	☐	☐	☐	☐

16. **My child notices if [Cat name] is hungry or thirsty**

Almost Never	Rarely	Sometimes	Very Often	Always or Almost Always
☐	☐	☐	☐	☐

17. **It would be upsetting for my child if [Cat name] died**

Definitely Not True	Not True	Partially True	True	Definitely True
☐	☐	☐	☐	☐

18. My child wishes they could always be with [Cat name]

Definitely Not True	Not True	Partially True	True	Definitely True
☐	☐	☐	☐	☐

19. I have observed many special moments between my child and [Cat name]

Definitely Not True	Not True	Partially True	True	Definitely True
☐	☐	☐	☐	☐

20. My child checks that [Cat name] is safe in bed or settled at night

Almost Never	Rarely	Sometimes	Very Often	Always or Almost Always
☐	☐	☐	☐	☐

21. My child provides water for [Cat name]

Almost Never	Rarely	Sometimes	Very Often	Always or Almost Always
☐	☐	☐	☐	☐

22. My child cleans the litter box

Almost Never	Rarely	Sometimes	Very Often	Always or Almost Always
☐	☐	☐	☐	☐

23. [Cat name] sleeps with my child at night

Definitely Not True	Not True	Partially True	True	Definitely True
☐	☐	☐	☐	☐

24. My child makes sure [Cat name] is safe when visitors are in the house or are around the pet

Almost Never	Rarely	Sometimes	Very Often	Always or Almost Always
☐	☐	☐	☐	☐

25. My child entertains or plays with [Cat name]

Definitely Not True	Not True	Partially True	True	Definitely True
☐	☐	☐	☐	☐

26. My child finds it difficult to balance their time between [Cat name] and other activi-ties (e.g., school, after school events, visiting friends)

Almost Never	Rarely	Sometimes	Very Often	Always or Almost Always
☐	☐	☐	☐	☐

27. **[Cat name]'s behaviour (or noise) bothers or irritates my child**

Definitely Not True	Not True	Partially True	True	Definitely True
☐	☐	☐	☐	☐

28. **My child feels overwhelmed by [Cat name]'s care needs**

Almost Never	Rarely	Sometimes	Very Often	Always or Almost Always
☐	☐	☐	☐	☐

29. **My child feels that [Cat name] stops them from doing things they want to do (e.g., visiting friends, travelling)**

Almost Never	Rarely	Sometimes	Very Often	Always or Almost Always
☐	☐	☐	☐	☐

Administration and Scoring Instructions for the C-CES

Each item is scored on a five-point scale, from 1 to 5. As specified below, four items should be reverse scored, such that a higher score indicates stronger relationships.

To calculate the Emotional Attachment subscale score, add the scores for items 1, 4, 5, 6, 8, 9, 12, 13, 14, 17, 18, 19, 20, 23, 24, and 25. Then divide by 16.

To calculate the Engagement subscale score, add the scores for items 2, 3, 7, 10, 11, 15, 16, 21, and 22. Then divide by 9.

To calculate the Challenges subscale score, reverse score items 26, 27, 28 and 29. Then add the scores and divide by 4.

To calculate the Total score, sum the three subscale scores.
