# Supplementary material for: Measuring Connections Between Children and Pets: Development of the Child–Dog Engagement Scale and Child–Cat Engagement Scale
Source: Animals (Basel). 2025 Jun 22;15(13):1845. doi: 10.3390/ani15131845 (PMC12248443; doi:10.3390/ani15131845)
Supplement: Supplementary file 1 [file animals-15-01845-s001.zip › File S3 - Frequency table 22.06.2025.pdf]

File S3: Response frequencies for the the C-DES and C-CES

Table S1. Response frequencies and percentages for final Child-Dog Engagement Scale items

Item	Definitely Not True *or* Almost Never (1)	Not True *or* Rarely (2)	Partially True *or* Sometimes (3)	True *or* Very Often (4)	Definitely True *or* Always/Almost Always (5)
I have observed many special moments between my child and [Pet name]	1 (0.6%)	1 (0.6%)	15 (9.0%)	55 (32.9%)	95 (56.9%)
My child likes to be near [Pet name] when relaxing (e.g., reading, watching TV)	2 (1.2%)	7 (4.2%)	24 (1.4%)	42 (25.1%)	92 (55.1%)
When my child is away from [Pet name] for an extended time, they miss them	1 (0.6%)	6 (3.6%)	15 (9.0%)	52 (31.1%)	93 (55.7%)
It would be upsetting for my child if [Pet name] died	0 (0.0%)	0 (0.0%)	4 (2.4%)	27 (16.2%)	136 (81.4%)
My child loves [Pet name]	0 (0.0%)	0 (0.0%)	3 (1.8%)	35 (21.0%)	129 (77.2%)
My child feels like [Pet name] helps them through tough times	2 (1.2%)	14 (8.4%)	38 (22.8%)	60 (35.9%)	53 (31.7%)
My child considers [Pet name] to be one of their best friends	2 (1.2%)	15 (9.0%)	20 (12.0%)	59 (35.3%)	71 (42.5%)
My child feels happy when interacting with [Pet name]	0 (0.0%)	1 (0.6%)	8 (4.8%)	51 (30.5%)	107 (64.1%)
My child likes to talk about [Pet name] with me and/or other people	1 (0.6%)	1 (0.6%)	18 (10.8%)	63 (37.7%)	84 (50.3%)
My child likes to show appropriate physical affection towards [Pet name] (e.g., gentle pats, hugs, or kisses)	1 (0.6%)	0 (0.0%)	18 (10.8%)	80 (47.9%)	68 (40.7%)
My child wishes they could always be with [Pet name]	2 (1.2%)	15 (9.0%)	32 (19.2%%)	61 (34.1%)	57 (34.1%)
My child thinks [Pet name] is just a pet [a]	6 (3.6%)	18 (10.8%)	33 (19.8%)	68 (40.7%)	42 (25.1%)
My child believes [Pet name] understands their [my child's] feelings or emotions	3 (1.8%)	6 (3.6%)	34 (20.4%)	74 (44.3%)	50 (29.9%)
My child cleans up inside after [Pet name]	43 (25.7%)	35 (21.0%)	48 (28.7%)	19 (11.4%)	22 (13.2%)
When necessary, my child gives [Pet name] medicine/vitamins	55 (32.9%)	33 (19.8%)	28 (16.8%)	31 (18.6%)	20 (12.0%)
My child cleans up outside after [Pet name]	55 (32.9%)	24 (14.4%)	44 (26.3%)	22 (13.2%)	22 (13.2%)
My child takes [Pet name] outside for toilet breaks	13 (7.8%)	19 (11.4%)	51 (30.5%)	48 (28.7%)	36 (21.6%)
My child washes or bathes [Pet name]	36 (21.6%)	34 (20.4%)	41 (24.6%)	34 (20.4%)	22 (13.2%)
My child provides water for [Pet name]	4 (2.4%)	10 (6.0%)	46 (27.5%)	60 (35.9%)	47 (28.1%)

My child helps with obedience training [Pet name], including informal training at home (e.g., teaching [Pet name] to sit)	13 (7.8%)	20 (12.0%)	53 (31.7%)	57 (34.1%)	24 (14.4%)
My child takes [Pet name] for visits outside the house (e.g., to other family members, school)	35 (21.0%)	21 (12.6%)	54 (32.3%)	36 (21.6%)	21 (12.6%)
My child notices if [Pet name] is hungry or thirsty	4 (2.4%)	22 (13.2%)	45 (26.9%)	49 (29.3%)	47 (21.81%)
My child brushes or grooms [Pet name]	14 (8.4%)	24 (14.4%)	58 (34.7%)	42 (25.1%)	29 (17.4%)
My child makes sure [Pet name] is safe when visitors are in the house or are around the pet	8 (4.8%)	21 (12.6%)	46 (27.5%)	60 (35.9%)	32 (19.2%)
My child takes [Pet name] for walk/exercise	9 (5.4%)	13 (7.8%)	42 (25.1%)	64 (38.3%)	39 (23.4%)
My child provides food for [Pet name]	3 (1.8%)	6 (3.6%)	60 (35.9%)	64 (38.3%)	34 (20.4%)
My child gives [Pet name] treats	1 (0.6%)	1 (0.6%)	39 (23.4%)	68 (40.7%)	58 (34.7%)
My child helps with teaching [Pet name] new tricks	17 (10.2%)	18 (10.8%)	51 (30.5%)	54 (32.2%)	27 (16.2%)
When necessary, my child goes to the veterinarian with [Pet name]	29 (17.4%)	37 (22.2%)	39 (23.4%)	30 (19.0%)	32 (19.2%)
My child notices if [Pet name] is sick	5 (3.0%)	14 (8.4%)	49 (29.3%)	55 (32.9%)	44 (26.3%)
My child takes photos or videos of [Pet name]	5 (3.0%)	15 (9.0%)	54 (32.3%)	58 (34.7%)	35 (21.0%)
My child checks that [Pet name] is safe in bed or settled at night	16 (9.6%)	16 (9.6%)	54 (32.2%)	55 (32.9%)	26 (15.6%)
[Pet name] sleeps with my child at night	64 (38.3%)	20 (12.0%)	47 (28.1%)	25 (15.0%)	11 (6.6%)
My child is respectful of [Pet name]’s space (e.g., approaching calmly, giving the animal plenty of room)	1 (0.6%)	9 (5.4%)	33 (19.8%)	84 (50.3%)	40 (24.0%)
My child feels that [Pet name] stops them from doing things they want to do (e.g., visiting friends, travelling) [a]	1 (0.6%)	9 (5.4%)	17 (10.2%)	45 (26.9%)	95 (56.9%)
My child feels overwhelmed by [Pet name]'s care needs [a]	5 (3.0%)	9 (5.4%)	24 (14.4%)	45 (26.9%)	84 (50.3%)
My child finds it difficult to balance their time between [Pet name] and other activities (e.g., school, after school events, visiting friends) [a]	6 (3.6%)	13 (7.8%)	28 (16.8%)	73 (43.7%)	47 (28.1%)
My child gets too rough with [Pet name] during play or when showing affection [a]	1 (0.6%)	9 (5.4%)	33 (19.8%)	48 (28.7%)	76 (45.5%)

[a] = item is reverse scored

Table 2. Response frequencies and percentages for final Child-Cat Engagement Scale items

Item	Definitely Not True *or* Almost Never (1)	Not True *or* Rarely (2)	Partially True *or* Sometimes (3)	True *or* Very Often (4)	Definitely True *or* Always/Almost Always (5)
I have observed many special moments between my child and [Pet name]	0 (0.0%)	2 (1.3%)	15 (9.9%)	50 (32.9%)	85 (55.9%)
My child likes to talk about [Pet name] with me and/or other people	1 (0.7%)	2 (1.3%)	15 (9.9%)	61 (40.1%)	73 (48.0%
My child considers [Pet name] to be one of their best friends	3 (2.0%)	11 (7.2%)	33 (21.7%)	51 (33.6%)	54 (35.5%)
When my child is away from [Pet name] for an extended time, they miss them	1 (0.7%)	6 (3.9%)	16 (10.5%)	57 (37.5)	72 (47.4%)
My child likes to be near [Pet name] when relaxing (e.g., reading, watching TV)	0 (0.0%)	3 (2.0%)	10 (6.6%)	60 (39.5%)	79 (52.0%)
My child loves [Pet name]	0 (0.0%)	0 (0.0%)	3 (2.0%)	29 (19.1%)	120 (78.9%)
My child wishes they could always be with [Pet name]	3 (2.0%)	7 (4.6%)	38 (25.0%)	60 (39.5%)	44 (28.9%)
My child feels like [Pet name] helps them through tough times	2 (1.3%)	9 (5.9%)	46 (30.3%)	60 (39.5%)	35 (23.0%)
My child believes [Pet name] understands their [my child's] feelings or emotions	0 (0.0%)	17 (11.2%)	38 (35.0%)	59 (38.8%)	38 (25.0%)
My child likes to show appropriate physical affection towards [Pet name] (e.g., gentle pats, hugs, or kisses)	0 (0.0%)	3 (2.0%)	13 (8.6%)	58 (38.2%)	78 (51.3%)
It would be upsetting for my child if [Pet name] died	0 (0.0%)	1 (0.7%)	3 (2.0%)	15 (9.9%)	133 (87.5%)
My child checks that [Pet name] is safe in bed or settled at night	19 (12.5%)	14 (9.2%)	46 (30.3%)	51 (33.6%)	22 (14.5%)
My child entertains or plays with [Pet name]	0 (0.0%)	2 (1.3%)	13 (8.6%)	74 (48.7%)	63 (41.4%)
[Pet name] sleeps with my child at night	33 (21.7%)	27 (17.8%)	37 (24.3%)	33 (21.7%)	22 (14.5%)
My child feels happy when interacting with [Pet name]	0 (0.0%)	0 (0.0%)	6 (3.9%)	53 (34.9%)	93 (61.2%)
My child makes sure [Pet name] is safe when visitors are in the house or are around the pet	30 (19.7%)	23 (15.1%)	51 (33.6%)	29 (19.1%)	19 (12.5%)
My child provides water for [Pet name]	1 (0.7%)	11 (7.2%)	42 (27.6%)	48 (31.6%)	50 (32.9%)
My child notices if [Pet name] is sick	20 (13.2%)	18 (11.8%)	72 (47.4%)	42 (27.6%)	0 (0.0%)
My child provides food for [Pet name]	11 (7.2%)	24 (15.8%)	49 (32.2%)	12 (7.9%)	56 (36.8%)

When necessary, my child gives [Pet name] medicine/vitamins	77 (50.7%)	32 (21.1%)	25 (16.4%)	9 (5.9%)	9 (5.9%)
When necessary, my child goes to the veterinarian with [Pet name]	37 (24.3%)	33 (21.7%)	37 (24.3%)	45 (29.6%)	0 (0.0%)
My child cleans up after [Pet name]	27 (17.8%)	36 (23.7%)	43 (28.3%)	36 (23.7%)	10 (6.6%)
My child gives [Pet name] treats	1 (0.7%)	3 (2.0%)	35 (23.0%)	63 (41.4%)	50 (32.9%)
My child notices if [Pet name] is hungry or thirsty	4 (2.6%)	5 (3.3%)	40 (26.3%)	61 (40.1%)	42 (27.6%)
My child cleans the litter box	63 (41.4%)	22 (14.5%)	30 (19.7%)	20 (13.2%)	17 (11.2%)
My child feels overwhelmed by [Pet name]'s care needs [a]	2 (1.3%)	10 (6.6%)	22 (14.5%)	30 (19.7%)	88 (57.9%)
My child finds it difficult to balance their time between [Pet name] and other activities (e.g., school, after school events, visiting friends) [a]	3 (2.0%)	9 (5.9%)	31 (20.4%)	57 (37.5%)	52 (34.2%)
My child feels that [Pet name] stops them from doing things they want to do (e.g., visiting friends, travelling) [a]	1 (0.7%)	5 (3.3%)	10 (6.6%)	29 (19.1%)	107 (70.4%)
[Pet name]'s behaviour (or noise) bothers or irritates my child [a]	1 (0.7%)	7 (4.6%)	14 (9.2%)	60 (39.5%)	70 (46.1%)

[a] = item is reverse scored
